# AI in Neurological Health Care: Qualitative Study of Patient and Public Perceptions

**DOI:** 10.2196/87806

**Published:** 2026-09-24

**Authors:** Tina Bedenik, Orna Fennelly, Kathleen Bennett

**Affiliations:** 1 Data Science Centre School of Population Health RCSI University of Medicine and Health Sciences Dublin, Leinster Ireland; 2 FutureNeuro Research Ireland Centre for Translational Brain Science RCSI University of Medicine and Health Sciences Dublin, Leinster Ireland; 3 Digital Health Department Children’s Health Ireland Dublin, Leinster Ireland; 4 School of Public Health, Physiotherapy and Sports Science University College Dublin Dublin, Leinster Ireland

**Keywords:** AI, artificial intelligence, clinical decision-making, health data, Ireland, qualitative research

## Abstract

**Background:**

Globally, health systems face increasing challenges due to improved life expectancy, rising levels of disease, greater expectations for health care, and growing expenditure. AI-based technologies are increasingly used to support clinical decision-making and contribute to effective delivery of care. AI has significant potential in terms of diagnostics and treatment for preventive and personalized medicine, including progressive, nonprogressive, or neurodivergent conditions affecting the brain, spinal cord, and nerves. Although AI has been extensively applied to several areas of health, its application to neurological conditions remains limited.

**Objective:**

This qualitative study explores the perspectives of patients and the public impacted by neurological conditions on the use of AI in health care in Ireland. It focused on the benefits and concerns regarding AI implementation to inform effective and safe use of AI in clinical decision-making.

**Methods:**

We conducted a qualitative cross-sectional study using focus groups of patients and the public (family members). A combination of purposive and snowball sampling was used in participant recruitment. The sample consisted of 15 participants involved in 2 focus groups, most commonly with conditions including early-onset Parkinson disease, epilepsy, and multiple sclerosis. Qualitative data were transcribed, and thematic data analysis was undertaken using NVivo (version 12; Lumivero) software.

**Results:**

The main benefits of AI in clinical decision-making included improved diagnostics and treatment for neurological health care, increased patient involvement in care and decision-making, and resource efficiency. Participants believed that AI-powered systems could assist with information collation and decision-making, and AI could be an aid in simple repetitive tasks, allowing clinicians more time for clinical work. The concerns included preparedness of the national health data ecosystem, harm resulting from AI application, and overreliance on AI resulting in deskilling. Participants were generally supportive of AI in clinical decision-making; however, their support was conditional on a stringent regulatory framework and human supervision over AI use.

**Conclusions:**

Neurological conditions have a significant impact on the lives of patients and their families, with large resource implications for the health care system. AI has the potential to contribute to significant benefits, such as improved diagnostics, resource efficiency, novel insights, and improved provision of health care. However, the health care system needs to focus on the readiness and availability of health data. This study highlights the importance of preparing health systems for the expansion of AI systems, including supporting clinicians and patients in the appropriate and safe implementation of AI in health care.

## Introduction

Globally, health systems face increasing challenges with more demand for health care due to aging populations, improved life expectancy, rising levels of disease, greater demand for and expectation of health care, and growing expenditure. Decision support tools, including AI-based technologies, are increasingly being used to support clinical decision-making in health care and contribute to efficiencies and effective delivery of care [1]. An AI system refers to “a machine-based system that is designed to operate with varying levels of autonomy and that may exhibit adaptiveness after deployment, and that, for explicit or implicit objectives, infers, from the input it receives, how to generate outputs such as predictions, content, recommendations, or decisions that can influence physical or virtual environments” [2]. Different AI technologies have been implemented across health care. Clinical decision support systems (CDSs), which are augmented intelligence tools to support or complement decision-making, are commonly based on supervised and unsupervised machine learning and deep learning [3,4]. More recently, other tools have been incorporated into clinical decision-making, for example, in areas such as radiology for image analysis and in risk prediction, and often in the context of multimodal data [5]. Other tools support more resource efficiencies, such as conversational agents (eg, chatbots and speech recognition), based on machine learning and natural language processing [6], and large language models, including widely available tools such as ChatGPT [7].

AI systems have the potential to improve and transform how health care is delivered, ultimately leading to efficiencies and better health outcomes. In a systematic literature review of AI in health care, Ali et al [8] examined AI-enabled health care benefits, challenges, methodologies, and functionalities. The findings suggest that AI continues to outperform humans in specific aspects of medical and administrative processes related to accuracy, efficiency, and timely execution. Benefits for patients in diagnosis, treatment, and health monitoring for chronic conditions were identified. However, there were also concerns around patient safety, data integration, and legal, and privacy issues when used in care management. Previous evidence on the perspectives of patients, informal caregivers, and health care professionals (HCPs) on the use of AI in diagnostics and management in health care generally focuses on the limitations, potential benefits, and ethical and social aspects, but the evidence is mixed. In a review of barriers and facilitators influencing HCPs’ acceptance of AI in the hospital setting, the findings on perceptions of the effects of AI on error detection or alert sensitivity were heterogeneous, whereas limiting factors of loss of autonomy and integration of AI into clinical workflows were consistently reported [9]. A qualitative meta-synthesis of 12 studies on public perception of AI in health care found that the public acknowledged the advantages of medical AI; however, there were also concerns regarding ethical and legal issues [10]. The public perspective of any new digital implementation in health care is critical to successful implementation and effective adoption. Therefore, it is crucial that research further investigates public views on AI use in diagnosis, management, and care.

AI has been extensively applied to several areas of health care, including noncommunicable diseases such as cancer and cardiovascular disease, but it has had limited application in neurological conditions [11]. Due to the often progressive nature of neurological conditions and the wide range of symptoms, including physical, cognitive, emotional, and behavioral, patients and carers affected by such conditions can provide a unique perspective on how AI systems could be used to help manage conditions such as dementia, epilepsy, multiple sclerosis, and motor neuron disease. Diagnosis and management of these conditions often require multimodal data (ie, images, electroencephalography, and clinical information). As AI has been shown to integrate diverse types of patient data [5], it offers a powerful approach to advancing personalized medicine. For example, in dementia care, AI with multimodal data is improving differential diagnosis and early detection, informing disease progression and patient stratification for clinical trials [12]. However, qualitative and quantitative evidence on perspectives of AI in health care related to neurological conditions is lacking.

Therefore, this qualitative study aims to explore the perspectives of patients and the public impacted by neurological conditions on the use of AI in neurological health care. The goal of this study is to inform the effective and safe use of AI in clinical decision-making, with a specific focus on the benefits and concerns regarding AI implementation. Specifically, our research question was to ask what the perspectives of patients and the public are on the use of AI in the diagnosis, management, and care of neurological conditions.

## Methods

### Study Design

A qualitative cross-sectional study of the perspectives and expectations of patients and the public toward the application of AI in neurological health care in Ireland was conducted using focus group discussions. The conduct and reporting of this study followed the Consolidated Criteria for Reporting Qualitative Research (COREQ) guidelines [13].

### Research Team and Roles

The primary researcher on this project was TB, a senior postdoctoral fellow, who has 10 years of experience in conducting qualitative research. The principal investigator on the project was KB, a professor of biostatistics, with over 30 years of experience in undertaking medical research. Laura Brady, previously appointed as the digital health innovation lead, assisted with study design and recruitment, and research assistant Karen Fowler assisted during data collection and analysis. In an effort to reduce potential researcher bias due to backgrounds, knowledge, and specialization, the study protocol and supporting materials, such as the topic guide, were developed in a collaborative manner by the research team, and any differences in opinion were discussed and resolved through consensus. In addition, 1 transcript was coded by 2 members of the research team, and any differences in coding were explored to facilitate greater validity of the findings. In addition, the COREQ guidelines [13] ensured methodological rigor.

### Participant Recruitment

The study included patients with neurological conditions and members of the public whose family members were impacted by neurological conditions, such as parents of children, or who were otherwise involved in this field through work or advocacy. We have combined patients and the public into one group, as neurological conditions have a significant impact on the lives of patients, their families, and wider society. Only adults and those residing in Ireland were included in the study. A combination of purposive and snowball sampling methods was used in participant recruitment. Participant recruitment included those receiving health care for neurological disease, family members, and those interested in the application of AI in clinical decision-making. Procedures for recruitment involved the following: social media posts, for example, on LinkedIn, with an advertisement for the study; a news story advertising the study with known patient organizations such as Epilepsy Ireland; and snowballing of existing participants. The FutureNeuro Centre for Translational Brain Research was actively involved in assisting with recruitment. Those willing to take part in the study contacted the research team. The researchers had no prior relationship with the participants. The participants knew the researchers’ names, their affiliation, and the objectives of the study. Once initial contact was established, they were approached by the lead researcher via email.

### Data Collection and Analysis

The data were collected through online focus groups in spring and summer 2025 via the Zoom platform (Zoom Video Communications). The focus groups were organized and facilitated by TB, and a research assistant took notes. There were 2 focus groups with 7 and 8 participants, respectively, for approximately 2 hours each. Nobody else was present during data collection besides the participants and the researchers. A semistructured approach was used, and discussions were guided by a topic guide (Multimedia Appendix 1), informed by the literature review and developed through discussions between TB, KB, and Laura Brady. A pilot one-to-one online interview was conducted with a female patient with early-onset Parkinson disease. Based on the pilot interview and feedback from the participant, the topic guide was refined. To facilitate an informed discussion, participants were given a definition of AI in the participant information leaflet (Multimedia Appendix 2) and asked to watch a short video by The Economist titled “How AI Can Make Healthcare Better” [14] before the scheduled focus group, which provided an overview of the application of AI in health care, including the benefits and risks. As this was an exploratory qualitative study, our approach was to conduct an in-depth, localized exploration rather than generalization. We aimed for information sufficiency, completing focus group discussions once all questions had been answered. The focus groups were audio-recorded and transcribed verbatim, with all identifying markers removed prior to analysis.

Thematic data analysis was undertaken with the support of NVivo (version 12; Lumivero). TB coded the qualitative data and created the coding index, which was discussed with KB. One transcript was coded independently by TB and KF, and the results were compared. The analysis was driven by the data, where recurring patterns of meaning were sought in the data to generate themes, rather than relying on a preexisting coding framework [15,16]. The 6 phases of reflexive thematic analysis were followed: familiarization with the data, generating initial codes, searching for themes, reviewing themes, defining and naming themes, and producing the report. Considering that thematic analysis is not a linear process, movement back and forth between these 6 phases occurred. In addition, it has been argued that thematic analysis is a family of methods, rather than there being a standardized approach to conducting thematic analysis [17], and therefore elements of both deductive and inductive analysis were incorporated. Specifically, data analysis commenced deductively, with all data classified under four broad categories corresponding to the topic guide: (1) clinical decision-making, (2) benefits of AI application, (3) risks and concerns with AI application, and (4) regulatory framework. Subsequently, the analysis continued in an inductive way, and codes and subcodes were developed, reviewed, organized, and consolidated. The central focus was to juxtapose the benefits of AI application against the concerns. The themes and subthemes belonging to these 2 categories were selected due to the frequency with which they occurred in the data. Their frequency was determined by the number of references in the dataset, and themes and subthemes with the highest number of references were chosen for this article. Quotes have been provided to accompany narrative descriptions and support the findings, and each quotation was accompanied by the participant number.

### Ethical Considerations

Ethical approval was granted by the RCSI University of Medicine and Health Sciences Research Ethics Committee (REC202408016). Written informed consent was obtained from all participants prior to any data collection. It was indicated in the participant information leaflet that participants could request the transcript of the focus group and make changes pertaining to their statements; however, no participant made this request. The participants were not asked to provide feedback on the findings. To protect participants’ identities, demographic data were reported only in aggregate form, and all identifying information was removed prior to analysis. Participants in the patients and public group received an honorarium (voucher) for their contribution.

## Results

### Participant Demographics

The sample consisted of 15 participants. A total of 8 females and 7 males participated in the study, with the majority of participants aged between 35 and 54 years. All participants identified as Irish. Eleven participants had a third-level degree, 6 of whom had a postgraduate degree, and half of the participants were employed or self-employed. Eleven participants reported having a neurological condition, and 4 participants had a family member impacted by a neurological condition, for instance, a child. The most common conditions affecting the patients and/or their family members were early-onset Parkinson disease, epilepsy, Parkinson disease, and multiple sclerosis, while other conditions included Huntington disease, functional neurological disorder, dystonia, spina bifida, cerebral palsy, new daily persistent headache, and migraines. Some participants or participants’ children had more than 1 neurological condition.

### Themes and Subthemes

#### Overview

The results focused on 2 distinct categories: benefits and concerns regarding AI application in neurological health care in Ireland, and themes and subthemes within these 2 broad categories were explored. The benefits of AI application include the following themes: (1) improved diagnostics and treatment for neurological health care, (2) increased patient involvement in care and decision-making, and (3) resource efficiency. The concerns with AI deployment encompass the following themes: (1) preparedness of the national health data ecosystem, (2) harm resulting from AI application, and (3) overreliance on AI and deskilling. In addition to the aforementioned themes, other benefits and concerns regarding AI use were identified, albeit less frequently, which is why they were not included in this paper.

#### Benefits of AI Application

##### Overview

In both groups, the participants expressed their hope, optimism, and enthusiasm regarding the benefits of AI application in neurological health care and its potential to enhance patient experiences and outcomes. The benefits included improved diagnostics and treatment, increased patient involvement, and resource efficiency.

##### Improved Diagnostics and Treatment for Neurological Health Care

###### Overview

This theme encompassed diagnostic speed and the production of novel insights through AI deployment, described below in more detail.

###### Diagnostic Speed

The participants agreed that AI could be a “fantastic tool to speed up the whole system” (P7) and assist with reading scans, reviewing images, and reaching a more rapid diagnosis, which is of significant importance in conditions such as brain tumors, where early detection is paramount. They also highlighted that AI implementation could be vital in Ireland, given that human life expectancy is increasing, while the country is experiencing a loss of radiologists due to migration.

The fact that the savings can be so dramatic in terms of a human being going through scans and possibly making mistakes, to a computer doing it in a matter of weeks or months and having very accurate results. It's just phenomenal what can be done – it's like a new world out there!

P9

Many of the patients reflected on their lengthy journey to reach a diagnosis, which in turn impacted their health and quality of life. Some recollected that even when they had “a label” (P12), they did not necessarily have the right treatment to alleviate the condition. However, they believed that AI could facilitate a fast diagnosis, which is helpful even if an adequate intervention is not readily available. This is because an early diagnosis enables patients to enter a community of those impacted by the same condition, and such social support may be part of the treatment, too.

We went through a ten-year period of getting a diagnosis for our son. The earlier you can get that and know what to expect [the better]. And [to] be part of a community, even though there’s no treatment, and it's not actionable. But having that community makes a huge difference, because then you can learn from each other.

P4

The support for using AI to assist with diagnostics was strong, albeit conditional. While the participants agreed that AI is a “brilliant companion tool for the doctors” (P4), they also advocated for a hybrid or augmented approach wherein HCPs review scans read by AI until the technology had been quality assured. They also contended that AI may help to expedite clinical trials and assist with the more rapid introduction of medication to the national market. This in turn could make the drug production process more cost-effective, and drugs more accessible and tailored to patients’ needs, with implications for the treatment of neurological conditions.

In order for a new drug to come to market you are talking about ten years for them picking a drug that could be tested as a candidate, and then to go through clinical trials. And by using modelling… The talk of the future is not just about medicine, it's precision medicine. And getting a drug that suits the patient that’s based on your particular genetic or physiological make-up.

P2

###### Production of Novel Insights

Another dimension of improved diagnostics was related to novel insights generated by and through AI. The participants believed that AI-powered systems may have the propensity to make connections that escape the human mind and make links that HCPs may not have considered. For instance, they suggested that AI algorithms could be useful in exploring how coexisting conditions interact.

I was going to a respiratory consultant, a urologist, a gastro person, whereas AI might be able to flag ‘Hang on a second, this patient is going to all these [consultants], and they didn’t see it as part of an overall syndrome.’ So, I was being treated for everything individually, rather than someone putting it together that this is actually EDS [Ehlers-Danlos syndromes] causing all the [issues].

P14

It was highlighted that many people with a neurological condition may have another medical condition. For instance, one participant (P8) stated that “a quarter of those with a functional neurological disorder (FND) have another neurological condition,” and suggested that these may be dystonia, multiple sclerosis, or Parkinson disease. With coexisting conditions, it may be difficult to differentiate between symptoms and treatments, and participants believed that AI represents a “massive opportunity” (P8) in that regard. Further, they thought that AI algorithms may explain how different medications interact and decrease the risk of adverse drug reactions. One patient reflected on what they called a “scary” experience.

I had an awful experience where a junior doctor prescribed a painkiller for me. And then as I went home, he rang and [left] five voice messages on my phone saying ‘Don’t take it, it actually will cause serotonin syndrome’, which would have potentially killed me. But that’s where AI could be helpful as long as there’s proper information shared within teams.

P9

In addition, the participants thought that AI algorithms may produce novel insights with implications not only for diagnosis and treatment but also for quality of life (QoL). It was highlighted that HCPs sometimes differentiate between neurological conditions that they treat, and QoL remains untreated. The participants wondered whether AI could be used not only as a means of producing new insights regarding the actual neurological condition, but also as a tool to generate helpful information about improving their QoL.

You might be living with the diagnosis for twenty or thirty years, but it is about QOL stuff, it is about different options, it is about innovation. That’s the real benefit of it, it's what we want. They talk to you almost as if you should be happy - you got a diagnosis, isn’t that great! If your QOL gets worse, what happens then?!

P5

The participants believed that AI would help provide more holistic care and facilitate improved QoL for patients with neurological conditions.

##### Increased Patient Involvement in Care and Decision-Making

###### Overview

The theme of increased patient involvement in care and decision-making focused on using AI as a means of patient empowerment and building better, more inclusive relationships with HCPs.

###### Patient Empowerment

Another benefit of AI use was the personalized support provided by AI-powered devices that may increase patient independence and self-governance, and thereby empower them. A common sentiment experienced by the patients was that of isolation. They believed that AI could provide emotional support and alleviate loneliness, so that the patients “aren’t going through this on their own” (P12). In other words, the patients described how they often felt left to their own devices once they received a diagnosis, and they expressed hope that the adoption of AI could support them in managing their health condition better. This would inevitably empower them.

I'm in the very early stages, so you are left to your own devices, and it's a lot of self-learning. I'd be hoping AI maybe could get something for someone with my symptoms, and I'd be able to follow that path, if that path is working.

P11

Some patients used AI applications such as ChatGPT to get motivated for the day, or in situations when human support was not readily available. In addition, they believed that AI-powered devices can bring together people impacted by a similar condition to build a support community.

Actually you sit in a period for, I did for a couple of years, where just waking up and being, living, and breathing for that day was victory enough. So, is there a role for AI to play in terms of the interconnectivity beyond just the consultant visit.

P12

It was reported that many patients used AI for structuring documents, monitoring their exercise, and getting help with outdoor walking through the use of beats and music. A significant benefit of AI lies in its ability to provide reminders about medication for patients with cognitive impairment issues.

The patient doesn’t always remember things correctly. A big one is ‘Did you take your medication or not take your medication?’ You might have these little tricks of turning the bottle a certain way, but you can still get that mixed up. So, just in terms of [AI] helping the patient manage different things when you have a complex condition.

P14

Such use of AI increases patient awareness and the ability to manage their condition more independently. However, some patients also use AI for advice on medication unbeknownst to their HCP. One patient with Parkinson disease recalled a situation in which they struggled with involuntary movement, and they turned to AI-powered systems for advice.

I was going to the neurologist, and in the end, I asked AI what should I do with this involuntary movement. [AI] suggested that I cut down on one of my drugs, Viropixin, which is very harsh. So, I cut it down in half and I asked the neurologist would that be okay. And he said ‘Yeah, we’ll try that as an experiment.’ So, at the end of the year that was fine, my involuntary movement was gone.

P10

Although the outcome was positive, this situation illustrated the complexities of independent patient use of AI that can easily become counterproductive, calling for a considered and cautious approach.

###### Building Partnerships

Another aspect of increased patient involvement through AI use lies in building stronger and more inclusive relationships with HCPs. The participants believed that AI may help bring patients into the decision-making process and have a greater input into their care and that of others.

Very often when you go to the appointments, they don’t tell you exactly what they are looking for. If they could give you a list in advance of things to monitor, and then [you] give them that list at the end of three or four months, that could really help in treatment.

P5

In other words, the participants wondered whether AI could be deployed as a tool to initiate a more active patient role and to foster greater engagement between the patient and their consultant and a flow of information. Some patients used the term “partnership” (P9) to describe the relationship with their consultant, or alternatively, what that relationship should look like. However, they were cognizant of the power discrepancies between patients and consultants. The patients expressed their wish for greater involvement in decision-making processes and increased knowledge about their condition. They believed that this could be enabled through the collection and monitoring of personal health data through AI.

There’s a greater role for the patient in maintaining their records and their own data, and taking control of their data. They are the data controller. I am the best person to assist in a diagnosis. I know my own body better than anybody.

P5

The participants concluded that leaving decision-making with the clinician was a “waste of time” (P5), considering that every patient is unique and therefore best placed to advise on their own care.

##### Resource Efficiency

The participants agreed that AI could be deployed for a range of tasks undertaken by HCPs that are straightforward and time-consuming.

The benefit plus the risk is the aspect of freeing up physician’s time in order to enhance and treat the experience. Things that are fundamentally secure, in terms of diagnosis and pathways, that are not high risk, AI can help support that in order to alleviate some of the day-to-day burden the consultants are facing.

P15

Specifically, they expressed dissatisfaction with the time assigned for consultations and appointments, which they deemed insufficient, and wondered whether AI could automate some tasks to maximize the time for interpersonal interaction.

We have only one visit in the year, so we’ve only fifteen minutes to get everything covered. That is not really possible. Maybe AI could be used to make the most of the fifteen minutes, get better feedback.

P13

The participants also believed that the use of AI will be driven by the needs of HCPs rather than those of patients, in order to address the challenges experienced in the health care sector, such as appointment time.

#### Concerns With AI Deployment

##### Overview

Although the participants’ perspectives toward AI use in health care were positive, significant areas of concern were identified. These were related to the preparedness of the national health data ecosystem for the application of AI, harm resulting from AI use, and overreliance on AI and deskilling.

##### Preparedness of the National Health Data Ecosystem

#### Overview

The theme of preparedness of the national health data ecosystem for AI application focused on the quality of health data used by AI-powered systems, privacy and security issues, as well as the national health IT system, which participants deemed fragmented and underdeveloped.

#### Health Data Quality and Protection

The participants were concerned about the quality of health data used by AI-powered systems, including data inconsistencies and how these may impact the accuracy of outputs. Fears were expressed about incorrect and outdated information being used by AI, resulting in inaccurate diagnoses.

If you don’t put the right information in, and listen to the patient and their particular subset of symptoms, then you are not going to get something that is reproduceable. And that accountability… because I wouldn't like somebody to turn around and go ‘Yeah gosh, we didn’t spot that because we just let the computer read it’.

P1

They also raised questions about the quality of data, compliance with data protection laws, and the need to collect data from sources outside of Europe, provided that the data comes from a trusted source.

Whether it's compliant with GDPR is something that needs to be considered. Where they gather the information – is it only in Europe or do they scrape from everywhere around the world? So, within Europe, should the ChatGPT only take their information from Europe? If that’s the case, you could be losing a good resource.

P13

There was agreement that patients have a role to play regarding data use through educating themselves, keeping better records, and engaging with the health care system responsibly. In addition, the participants highlighted that the health data source, as well as the volume of data in existence, may influence algorithms and cause bias. This was of particular relevance given that the population in Ireland is becoming much more diverse, and this change needs to be reflected in datasets. They also believed that some neurological conditions are underrepresented, and data from patients with those conditions may be absent or underused by AI.

If you have a condition that is not of high interest, you are not going to make the headlines in peer-review publications. I find that there’s certain conditions that sit on that periphery. And I'm concerned about a programming aspect of AI, [that] a set of neurological conditions will be bypassed.

P12

Furthermore, fears were expressed about privacy being compromised should there be a disclosure or misuse of health data used by AI.

My daughter happened to be in hospital during COVID and during the hack, and it was just sad to see a hospital reduced to one working computer. And obviously the data [gets] compromised, so security is a huge issue. Because AI can fall into hands of people who can use AI to steal data.

P15

It was further mentioned that data accuracy goes hand in hand with data protection. Provided that their data was kept safe and secure, the patients were willing to share their data to advance science. However, the participants believed that not all health data are equally sensitive.

My concern is in the area of genomics. Say, if someone gets your DNA and then you are going to get this disease in your forties or fifties, and then your insurance is through the roof. Is it going to create a two-tier system in another way?

P14

#### Health IT System

Another highlighted issue revolved around the underdeveloped and fragmented national health IT system within which to implement AI in clinical decision-making. It was important to have IT systems and electronic health records (EHRs) in place for AI to be fully implemented or incorporated into clinical decision-making. The participants were primarily concerned about the general absence of national interoperable EHRs.

We don’t have a digitalised patient record. That in itself becomes just ginormous problematic. If you are lucky enough that you have built a relationship with your consultant, they may remember you. But they are looking at paper notes. I attend an annual appointment, and I have to start from the very beginning. When you have that fifteen-minute window you think ‘Could you not have read that?!’

P12

The lack of EHRs was felt to contribute to a lack of cross-team collaboration and disjointed patient care.

My son is seeing the neurologist and different consultants in different hospitals, and things aren’t joined up. I always have that fear that maybe AI decisions are being made without the full picture. That’s a big concern.

P4

In light of these issues, the participants believed that the implementation of AI across the Irish health care system requires time and significant effort. This will be further exacerbated, according to the participants, by the “conservative approach” (P4) that the health service is taking due to the potential risks posed by AI.

I don’t see this as being the magic switch – a roll out of AI and then all of a sudden, the system works more efficiently. I think it's certainly going to be a huge, staged process.

P15

The health service is taking a conservative approach probably because of the potential risk. But I don’t understand what assessment has taken place and what confidence levels there are, and how is it going to be monitored if it's implemented. I think that’s important in terms of any new things being implemented.

P4

##### Harm Resulting From AI Application

#### Overview

The theme harm resulting from AI application encompassed misuse of AI, both clinical and patient misuses, and errors associated with AI use, both of which may pose a risk to patients’ health.

#### Misuse of AI

The participants thought that a wider application of AI in health care may lead to more independent—yet unsupervised—patient use, posing a risk of patient AI misuse that can alter a patient’s trajectory.

It could be very easy to say ‘Well I don’t feel so great taking my full dose, maybe I will reduce it down on my own.’ I think there’s a certain level of risk that AI could create, and for patients to say ‘Oh, but AI now becomes my source of truth because it has bigger brains, plus my appointment is six months away and I'm struggling at the moment.’

P15

It was highlighted that unsupervised patient use of AI may partly stem from infrequent medical appointments, and patients are “left to [their] own devices” (P11). The participants also warned about the ethical dilemmas surrounding AI use, given that HCPs and the pharmaceutical industry may be driven by innovation and profit, which could give rise to possible clinical misuse.

If there are doctors [whose] future careers and income is also based on research and technological advancements, how do you protect from that? And the drug companies, there’s been examples of drugs that have been horrendously harmful and dangerous. Who is to say AI will be grossly misused by people to make money and cause incredible harm?

P9

In addition, it was underscored that patients with specific neurological conditions may turn to AI in times of desperation and be “lulled into spending a lot of money on something that [has] no credibility” (P8). To counter this, the patients called for a very stringent regulatory framework to guide how AI is governed within health care.

#### Errors Associated With AI Use

The participants were worried about the errors associated with AI use, with patients and the public expressing particular concern about incorrect diagnosis and treatment. One participant drew on their experience of utilizing a remote device to monitor their son’s epileptic seizures at night. This was a wrist-worn wearable that monitors a person’s movement to detect possible convulsive seizures.

It would send me an alert on my phone to get up. But you can get all kinds of false alerts. And then his seizure types change, so it was more suitable for picking up tonic-clonic [generalised seizure], but not for the different types of seizures. All of these tools are great, but you need that human interaction.

P4

They expressed concern and mistrust regarding the accuracy of outputs produced by AI, which may explain why participants’ support for AI adoption in health care was conditional.

##### Overreliance on AI and Deskilling

Finally, concerns were expressed about overreliance on AI, which may diminish clinicians’ relationships with patients and result in deskilling. In other words, the participants believed that AI could assist but not deliver health care.

[AI] shouldn't just be let loose. There’s a certain art to medicine and part of [it] is the human aspect and patient engagement. Some doctors don’t necessarily know how to talk to people. And if you hand everything over to AI there’s a real risk that those doctors would become even worse at talking to people.

P3

In addition to a loss of communication and interpersonal skills, it was underlined that overuse of AI may result in a loss of clinical skills among HCPs.

If you went into the shop and bought five things, you could tot it up in your head just like that. And that’s because numerical literacy was a thing. But since the advent of the calculator, numerical literacy has gone down. That’s one of the risks that you are facing. People will lose knowledge because they no longer use it.

P2

The participants believed that another risk posed by overreliance on AI lies in its producing outputs out of context. This is why they advocated for a hybrid approach with human oversight, which should come from HCPs as well as patients, when appropriate.

## Discussion

### Main Findings and Comparison With Previous Research

This study’s findings suggest that patients and the public were supportive of AI implementation in health care to inform clinical decision-making. However, optimism coexisted with fears about the preparedness of the Irish health system for AI adoption, as well as significant privacy and ethical concerns regarding health data use and sharing. Specifically, participants believed that AI implementation could lead to improved diagnostics and treatment, resulting in earlier disease detection and treatment, and that AI-powered systems could assist HCPs with information collation and decision-making. However, issues with data integration and impeded information flow were identified as weaknesses, as reported elsewhere [8]. Participants anticipated that AI could produce novel insights and illuminate how coexisting conditions or medications interact. Participants also believed that AI could facilitate increased patient involvement, as AI-powered systems provide a range of supports and enable patients to manage their conditions more independently. AI-powered devices may also bring patients and their families into the decision-making process and help build more inclusive relationships with clinicians. This is supported by earlier research by Young et al [18] that emphasizes a need for human supervision to enable safe patient-centered implementation of clinical applications of AI. Additionally, our study found that the main advantages of AI include increased accuracy and efficiency and patient involvement in care, which coexisted with risks and concerns about dependency on technology that may deteriorate clinicians’ skills. There was general agreement among participants that AI is useful for simple repetitive tasks, allowing clinicians more time for clinical work, as previously identified [18]. Concerns were raised about the national health data ecosystem, which participants described as fragmented, outdated, and ultimately unprepared for AI implementation. Concerns were also expressed about the quality and representativeness of data used by AI-powered systems, and participants were concerned about the safety of their data. In addition, participants had fears about the misuse of AI by HCPs and the pharmaceutical industry, as well as errors associated with AI use that could lead to incorrect diagnoses. The participants explicitly advocated for a hybrid approach with human supervision and clinical judgment and a stringent regulatory framework guiding the use of AI in health care.

Despite the limited evidence base on the application of AI in neurological health care, there are some generalizations from other studies that have examined AI in health care decision-making. Samhammer et al [19] found positive, albeit conditional, support for AI application in health care, while Flanagan et al [20] concluded that AI-assisted decision-making in primary care was considered with cautious optimism by patients with multiple long-term conditions. Similar to our study, these findings identified potential benefits in coordination and personalization of care, but concerns about privacy, fairness, and the risk of diminished human connection were also raised. Beets et al [21] conducted a US-based systematic review of 11 surveys of public perceptions of AI in health care and found that the public anticipates that medicine is an area where AI applications could be of benefit; however, there were also significant concerns regarding the application of AI in decision-making and the privacy of health data. The results also corroborate our previous findings, which examined challenges with the secondary use of health data, and reaffirmed that patients and the public are willing to share their data for altruistic reasons, provided that privacy and security are ensured [22]. In addition, our findings confirm that issues with health data quality, alongside a fragmented national health data ecosystem and a lack of system interoperability, represent significant barriers to reusing health data and to applying and training AI [23].

### Study Strengths and Limitations

The strengths of the study include ascertaining the views of patients and the public in relation to neurological conditions, for which there is limited evidence. Neurological conditions, especially those that are progressive, have a significant impact on the lives of those with the condition as well as their families and contribute to significant resource implications for the health care system [24]. Therefore, AI could bring significant benefits to this particular population, and it is important to explore the views of these patients and their families on the use of AI to inform future implementations. While this study was conducted in Ireland, with implications for the representativeness and generalizability of the findings internationally, it provides insight into the unique perspectives of a country undergoing an expansive digital transformation, as observed internationally.

Saturation was not intended in this study, as this was an exploratory qualitative study in an area in which little to no scientific research had been conducted to date in Ireland. The findings of this study provide evidence to inform debate about the application of AI in clinical decision-making, rather than to provide definitive answers. In addition, it has been argued that saturation is not consistent with the values and assumptions associated with thematic analysis, since judgments about when to cease collecting data are situated and subjective [25]. Therefore, the focus groups concluded once all questions had been answered, including any questions that may have been raised by participants. A limitation is that we did not differentiate between the use of AI by clinicians and its use by patients and the public. This was an exploratory study in a novel area, and we investigated perspectives on the use of AI in health care jointly, which may have had implications for the coding process.

Several recruitment strategies were used over a period of time to recruit patients and the public into our 2 focus groups. The participants interested in and consenting to participate were included in the 2 focus groups (15 in total). Due to the limited number of participants coming forward and the timeframe, we were not able to recruit additional patient and public focus groups. Any adjustments to the questions and conversations were made verbally during the focus group discussion. These did not involve adjusting the wording of the topic guide. The study aim was not to compare the perspectives of patients and others, but to consider the group perspectives as a whole. In addition, most of those involved in the focus groups were patients (11/15), with only 4 family members, which would limit any comparison between these participant groups. Finally, our sample consisted of educated individuals, several of whom were active in public and patient involvement and patient advocacy, also contributing to deeper perspectives. Similar to previous research, further exploration of the perspectives of underrepresented groups who may not have the same understanding or awareness of AI would be very informative [18].

### Study Implications and Future Research

While study participants referred to the many benefits of AI, concerns were also raised about the capacity of the Irish health care system infrastructure to support the successful implementation of AI tools. Irish health care lacks digital maturity compared to many other countries [26], but it is undertaking a digital journey under a new digital framework that references AI as key to its success [27]. This plan includes the rollout of digital platforms such as the National Shared Care Record, HSE Patient Health App, and interoperable EHRs across primary and secondary care. Therefore, this study comes at an opportune time to enable the incorporation of its findings into national health care plans to implement digital solutions, including AI systems. The findings should also help to mitigate some of the concerns raised in relation to siloed data and AI systems using limited data to support clinical decision-making. As well as the primary use of health care data, participants discussed the secondary use of their data and data altruism to enable the development of AI systems, including their cross-border application for those with rare neurological conditions. This demonstrates the potential support by Irish health care users for the implementation of the European Health Data Space (EHDS), which will support cross-border sharing of health data, including for the development of AI systems [28]. As a member of the European Union, Ireland will be implementing the EHDS and AI regulations over the coming years, which will require Ireland to advance its capabilities to support secure data sharing and the use of AI [2]. However, as noted by participants in this study, access to data alone will not result in the development of safe and effective AI systems, and additional European projects such as SHAIPED, which aims to improve access to data while prioritizing data protection, will be important in promoting the successful and safe implementation and adoption of AI systems in health care [29].

Another implication of this study was the need to educate and inform both HCPs and the public on using and interpreting the information provided by AI systems and/or technologies, avoiding overreliance and deskilling, to ensure that the potential benefits are derived from AI as opposed to the potential harms. As mentioned, there are many different types of AI systems that apply different approaches and require different types of data for validation. While the importance of including skills on the use of AI in HCP education has been highlighted in previous research, there is less research on the education of the public [30]. This study demonstrated that patients were using freely available AI tools to support the management of their conditions, but the participants in this study were well educated and understood the pitfalls of AI, including the transparency of the information provided by such tools. However, HCPs need to be prepared and aware that their patients are using AI and provide support on its safe use. Additionally, national support and communications on AI in health care for the public are recommended to ensure that patients use AI safely and effectively [31].

To build on the findings from this study, further research should explore the implementation of AI systems within a neurological setting. As well as the perspectives of patients and the public in neurological settings on the use of AI, minimal research appears to be available on the implementation of CDS tools using AI in neurological settings, with no neurological study population identified in a recent scoping review evaluating the implementation of CDS in inpatient settings [4]. As part of this implementation, it would be important to ensure that patients, the public, and HCPs trust the quality of the AI system to promote its adoption and use, as this study identified some distrust among this population, consistent with previous research [32]. Further research on HCP perspectives regarding this implementation would also be beneficial to support the findings on patient perspectives. While our original study also included a focus group of HCPs to gain their perspectives (not reported here), this was more challenging given the small number working in the field and the time required to conduct the qualitative study.

### Conclusion

This qualitative study found that patients and the public support the use of AI systems in neurological health care, but concerns were raised that require consideration to derive the full potential of AI. Benefits such as improved diagnostics, resource efficiency, and novel insights could result in better provision of health care to patients with neurological conditions. However, the health care system needs to focus on the available data, the readiness of the Irish data landscape, and avoiding the deskilling of HCPs. This study highlights the importance of preparing the health system for the expansion of AI systems, including supporting HCPs and patients in the appropriate and safe use of AI in health care.

## Data Availability

The datasets generated or analyzed during this study are available from the corresponding author upon reasonable request.

## Appendix

Multimedia Appendix 1 Topic guide for patients.

Multimedia Appendix 2 Participant information leaflet.

## Abbreviations

CDS: clinical decision support system
COREQ: Consolidated Criteria for Reporting Qualitative Research
EHDS: European Health Data Space
EHR: electronic health record
HCP: health care professional
QoL: quality of life
