# Supplementary material for: AI in Neurological Health Care: Qualitative Study of Patient and Public Perceptions
Source: J Med Internet Res. 2026 Sep 24;28:e87806. doi: 10.2196/87806 (PMC13650457; doi:10.2196/87806)
Supplement: Supplementary file 1 [file jmir_v28i1e87806_app1.docx]

**Collective intelligence towards the use of artificial intelligence (AI) to inform clinical decision-making**

**Topic guide: Patients**

CLINICAL DECISION-MAKING

Can you tell me about the current decision-making process with your HCPs?

Do you feel involved or informed when it comes to decisions around your care?

What are your experiences of using AI in clinical decision-making, or more generally healthcare?

What are your initial thoughts or feelings about the use of AI in clinical decision-making?

BENEFITS

Are there any particular benefits that you think AI could bring to your treatment or care?

What excites you the most about implementing AI in healthcare?

How do you envisage the future of AI in healthcare?

CONCERNS

What would you consider to be the main risks in using AI to assist with clinical decision-making?

Is there anything that scares or concerns you about the use of AI?

Have you ever had an experience of things going wrong with using AI-powered tools in healthcare?

How was the situation managed?

REGULATORY FRAMEWORK

What are your levels of trust towards using AI in healthcare?

How can we increase patient trust and acceptance of the AI-powered tools?

What should be the key principles of safe and responsible use of AI?

What should be the rights and responsibilities of patients in the context of AI application?

CONCLUSION

Is there anything you would like to add that we haven’t covered yet?
