# Supplementary material for: AI in Neurological Health Care: Qualitative Study of Patient and Public Perceptions
Source: J Med Internet Res. 2026 Sep 24;28:e87806. doi: 10.2196/87806 (PMC13650457; doi:10.2196/87806)
Supplement: Supplementary file 2 [file jmir_v28i1e87806_app3.docx]

**Participant Information Leaflet (Patients and public)**

**Collective intelligence towards use of artificial intelligence (AI) to inform clinical decision-making**

**Principal Investigator: Professor Kathleen Bennett**

Email: [kathleenebennett@rcsi.ie](mailto:kathleenebennett@rcsi.ie)

**Co-Investigator: Dr Tina Bedenik**

Email: [tinabedenik@rcsi.ie](mailto:tinabedenik@rcsi.ie)

**RCSI University of Medicine and Health Sciences**

School of Population Health, Data Science Centre, Beaux Lane House, Dublin 2

You are invited to take part in a research study to be carried out by Professor Kathleen Bennett, Principal Investigator (PI) and Dr Tina Bedenik, Senior Postdoctoral Fellow at the RCSI University of Medicine and Health Sciences, School of Population Health and the Science Foundation Ireland (SFI) FutureNeuro Centre.

Your participation in this study is on a voluntary basis. Please read the information provided below carefully before you decide whether you wish to take part in the study. You should clearly understand the risks and benefits of taking part, so that you can make a decision that is right for you. You do not have to take part in this study, and you can also change your mind about taking part in the study, even after the study has started. You do not have to give us a reason. Your decision will be received with respect and confidentiality.

We would be happy if you were able to accept this invitation to take part in this project. Your involvement would help us improve the quality of our health services. If you have any questions, please contact us - we are happy to help.

**Why is this study being done?**

Artificial intelligence (AI) can be described as “a broad scientific discipline with its roots in philosophy, mathematics and computer science that aims to understand and develop systems that display properties of intelligence”^[1]^. AI can be applied across different branches of healthcare, such as history taking, examination, imaging, therapeutics and prognosis. AI can be applied to help healthcare professionals make diagnoses and decide on treatment plans, which is known as clinical decision-making. This study explores the opportunities and challenges in deploying AI in clinical decision-making, and the mechanisms for ethical and trustworthy application of AI-powered tools in healthcare. The research will explore the perspectives, expectations and concerns of patients and members of the public, healthcare professionals and researchers about the use of AI in clinical decision-making.

**Who is organising and funding this study?**

This research is conducted by Professor Kathleen Bennett, Principal Investigator (PI) and Dr Tina Bedenik, Senior Postdoctoral Researcher at the RCSI Data Science Centre, School of Population Health. The study is funded by the Science Foundation Ireland (SFI).

**Why am I being asked to take part?**

You are being asked to take part in this research because you are an individual who may have encountered the use of AI in healthcare. We are interested in hearing about your experiences and views regarding such use. You can participate even if you do not know anything about this particular subject.

**How will the study be carried out?**

This study is a qualitative research that involves focus groups with patients and public, healthcare professionals and researchers about the use of AI to inform clinical decision-making. There will be separate focus groups for different categories of participants, and each individual group will meet once. The focus group will take place online via the Zoom™ platform. The focus group discussions are expected to last around one hour, but we may go beyond that time. We will explore your current perceptions of clinical decision-making processes; your perceptions of the main opportunities and challenges associated with the application of AI in healthcare and clinical decision-making, and your expectations regarding safe and ethical deployment of AI in healthcare.

This study has received ethical approval from the RCSI Research Ethics Committee and protocols are in place to safeguard your privacy and confidentiality. The focus group will be audio and video-recorded, and transcribed verbatim to enable data analysis. All the transcripts will initially be pseudonymised before full anonymisation. Pseudonymised means that all names, locations and other potentially identifiable data will be removed, and replaced with a participant ID in the transcripts, but a key to link this ID back to your information may be kept for any follow-up. The original transcripts, audio and video recordings will be destroyed. After the transcripts have been checked for accuracy and the data analysis has been completed, the key to identifying you will be deleted and the transcripts will be made anonymous.

**What will happen to me if I agree to take part?**

If you agree to take part, Dr Bedenik will contact you with further information about your assigned focus group, and to obtain informed consent. You will receive an electronic copy of the Consent Form. Basic demographic data will be collected when you complete the Consent Form. This includes information about your gender, age range, ethnicity and education. The focus group will be facilitated by Dr Bedenik. The focus group will be audio and video-recorded, and notes will be taken throughout.

You have the right to withdraw from the study and asked for your data not to be used. Your decision will be received with respect and confidentiality. You can withdraw from the study prior to, during and after the focus group, up until the point when data analysis commences. You can request to receive a copy of the transcript of the focus group, so that you can review the transcript and make changes.

**What are the benefits?**

The study will help provide evidence on how to ensure and create trust and transparency into the use of AI-powered tools in clinical decision-making. The information you provide will inform and promote a culture that supports a safe and ethical use of AI in healthcare, with implications for improved patient care and outcomes.

**What are the risks?**

There are no risks to you taking part in the study, only the additional time to take part in the focus group. Different mechanisms are in place to protect your privacy.

**Will it cost me anything to take part?**

It will not cost you anything to take part in this study.

**Is the study confidential?**

Your responses provided during the focus group discussions will be kept confidential. Your identity will not be revealed, and your details will not be shared with any third party.

This study has been approved by RCSI Research Ethics Committee. Professor Bennett is the data controller and Dr Bedenik is the data processor. The data are only accessible by Professor Bennett and Dr Bedenik. Several mechanisms are in place to safeguard your privacy. Following the focus group, the transcriptions will be pseudonymised. Pseudonymised means that all names, locations and other potentially identifiable data will be removed, and replaced with a participant ID in the transcripts but a key to link this ID back to your information may be kept for any follow-up. The original transcripts, audio and video recordings will be destroyed. The pseudonymised data and the identification key will be stored on the RCSI password-protected computers on a cloud-based OneDrive, which is an online location (‘cloud’) for storing documents.

The identification key will be kept separate from pseudonymised transcripts, and it will contain information that links individual participants to their participant ID. The identification key will managed by Dr Bedenik, and it will be deleted once the transcripts have been checked for accuracy and the data analysis has been completed. The anonymised transcripts will be considered for archiving through an online open access platform such as Zenodo or GitHub in order to facilitate FAIR (Findable, Accessible, Interoperable and Reusable) data access. You may disagree to the anonymised transcripts being made available, if you wish. You will not be identified in the final report or any future publication resulting from this study.

Your name and contact details will be retained until all data has been collected. It is expected data collection will not take longer than 6-9 months, at which point all contact information will be deleted. You will be able to access publications resulting from the study via the FutureNeuro webpage approx. 6-9 months after study completion.

**Data protection**

1. The data will be processed for the specific purpose of understanding how artificial intelligence (AI) may be used in the future to inform clinical decision-making.
2. The legal basis for processing the data is in relation to legitimate interests of the RCSI and for scientific research purposes.
3. Only the named researchers at RCSI (K.B and T.B.) will have access to the data.
4. The anonymised data will be archived through a platform such as Zenodo or GitHub in order to facilitate fair access. You can choose to disagree to this.
5. All transcribed focus group data will be initially pseudonymised and subsequently anonymised, and any potential to identify individuals will be minimised. There is negligible risk of any harm from processing the data.
6. You have a right to withdraw from the study prior to, during and after data collection, up until the point when data collection commences. You will be able to do this by contacting Dr Tina Bedenik at [tinabedenik@rcsi.ie](mailto:tinabedenik@rcsi.ie).
7. You have the right to lodge a complaint with the Data Protection Commissioner.
8. You have the right to access your data, and you can request a copy of the transcript following data collection.
9. You have the right to restrict or object to the processing of your personal data.
10. You have the right to have any inaccurate information about yourself corrected or deleted.
11. You have the right to have your personal data (i.e. the information you supplied throughout the focus group) deleted, prior to the analysis of the research findings.
12. You have the right to portability, which means to move your data from one controller to another in a readable format.
13. The data will not be used for profiling.
14. You have the right to object to automated processing including profiling if you wish.
15. The data will not be used for further purposes, other than those specified.
16. There will be no transfer of data outside of RCSI. Your data is pseudonymised and it will be stored on an encrypted drive on the RCSI password protected computer. Only fully anonymised data will be shared outside of RCSI.

**Where can I get further information?**

If you have any further questions about the study, please contact:

**Dr Tina Bedenik, Co-Investigator**

Email: [tinabedenik@rcsi.ie](mailto:tinabedenik@rcsi.ie)

**Professor Kathleen Bennett, PI**

Email: [kathleenebennett@rcsi.ie](mailto:kathleenebennett@rcsi.ie)

Mr Dónall King, Data Protection Officer

Email: dataprotection@rcsi.ie

1. Panch, T., Szolovits, P., & Atun, R. (2018). Artificial intelligence, machine learning and health systems. Journal of global health, 8(2), 020303. https://doi.org/10.7189/jogh.08.020303
